# Severe immune-related autoimmune hemolytic anemia induced by pembrolizumab: a case report with novel immunosuppressive strategy

**DOI:** 10.3389/fimmu.2026.1811425

**Published:** 2026-05-08

**Authors:** Qin Ye, Meng Li, Ping Zhou, Shan Huang, Ke Xie

**Affiliations:** 1 Department of Oncology, Sichuan Provincial People’s Hospital, School of Medicine, University of Electronic Science and Technology of China, Chengdu, Sichuan, China; 2 Department of Oncology, People’s Hospital of Xinjin, Chengdu, Sichuan, China

**Keywords:** autoimmune hemolytic anemia, cyclophosphamide, fluorouracil, immune checkpoint inhibitor, pembrolizumab

## Abstract

**Introduction:**

Immune checkpoint inhibitors (ICIs), including PD-1 inhibitors, have significantly improved survival outcomes in patients with melanoma; however, serious adverse events may occur as a consequence of immune dysregulation. Autoimmune hemolytic anemia (AIHA) is a rare but potentially life-threatening condition. While corticosteroids remain the cornerstone of AIHA management, some patients with severe anemia require additional immunosuppressive agents beyond steroids alone.

**Case report:**

A 52-year-old woman with anal canal malignant melanoma presented with severe generalized fatigue, lower back pain, and gross hematuria. She had received one cycle of pembrolizumab two weeks prior to presentation. She had severe anemia with reticulocytosis, elevated lactate dehydrogenase and bilirubin levels, and a positive direct Coombs test, and was diagnosed with severe AIHA secondary to pembrolizumab therapy. Despite initial treatment with corticosteroids and intravenous immunoglobulin (IVIG), hemolysis progressed. The condition was ultimately controlled with cyclophosphamide (CTX) and fluorouracil (5-FU) in addition to corticosteroids. Three months later, pembrolizumab was again administered and the resulting AIHA was managed with corticosteroids alone. The patient subsequently completed cycles 3–8 of pembrolizumab without further recurrence and achieved sustained tumor control.

**Conclusion:**

This case illustrates a novel immunosuppressive strategy for management of pembrolizumab-induced AIHA, a rare but serious hematologic adverse effect associated with immunotherapy. We describe the clinical presentation and report that the combination of CTX and 5-FU in addition to corticosteroids and IVIG was successful in treating severe AIHA. Further investigation in larger cohorts is warranted to validate this approach for intervention in AIHA. With appropriate and prompt management, safe rechallenge and continuation of ICIs may be feasible in selected patients.

## Introduction

Immune checkpoint inhibitors (ICIs), including programmed cell death protein 1/programmed death ligand 1 (PD-1/PD-L1) and cytotoxic T-lymphocyte–associated antigen 4 (CTLA-4) inhibitors, have become indispensable therapeutic agents for a wide range of malignancies, including melanoma, owing to their ability to significantly improve patient survival (1). ICIs enhance host antitumor immune responses through T-cell activation (2); however, this mechanism may also disrupt immune tolerance, leading to immune-related adverse events (irAEs) (3). Common irAEs involve the skin, liver, gastrointestinal tract, and endocrine system, manifesting as dermatitis, hepatitis, colitis, and endocrine disorders (4). Less frequently, hematologic irAEs may occur, including autoimmune hemolytic anemia (AIHA) (5) and immune thrombocytopenia (6), which can be severe and potentially life-threatening. Corticosteroids are the first choice for management of most irAEs, as they effectively suppress aberrant immune activation (7). However, some irAEs are too severe to be adequately managed with first-line corticosteroid therapy alone and require the addition of immunosuppressive agents.

We report the case of a patient with anal canal malignant melanoma who underwent surgical resection and subsequently developed AIHA after the first cycle of pembrolizumab therapy. Hemolysis progressed despite corticosteroid treatment and intravenous immunoglobulin (IVIG) but was ultimately controlled by the inclusion of cyclophosphamide (CTX) and fluorouracil (5-FU). A second episode of AIHA occurred following rechallenge with pembrolizumab three months later, but was effectively managed with corticosteroids alone, and the patient was able to complete cycles 3–8 of pembrolizumab with no AIHA recurrence. The success of this strategy incorporating CTX and 5-FU in addition to corticosteroids and IVIG for the management of severe AIHA warrants further validation in larger cohorts. Our experience underscores the importance of early recognition and accurate diagnosis of rare hematologic irAEs such as AIHA. With timely and appropriate management, safe rechallenge and continuation of ICIs may be feasible in selected patients.

## Case report

A 52-year-old woman presented to the emergency department with complaints of progressive fatigue, lower back pain, and gross hematuria. She reported that generalized weakness and lower back pain had begun two days prior to presentation and had progressively worsened. She denied fever, cough, chest pain, dyspnea, or any overt bleeding episodes. Her medical history was significant for recently diagnosed anal canal malignant melanoma, for which she had underwent surgical resection initially performed for presumed hemorrhoids. Postoperative immunohistochemical analysis demonstrated that the tumor cells were positive for S-100, HMB45, MelanA, and SOX-10, and negative for Dog-1, CD34, smooth muscle actin (SMA), desmin, cytokeratin (CK), CD117, and PGP9.5. STAT-6 showed partial positivity, and the Ki-67 proliferation index was approximately 70%. Molecular testing revealed no BRAF V600 mutation. Following surgery, immunotherapy was initiated with intravenous pembrolizumab, 100 mg. She had received one cycle of treatment, with the most recent dose administered 14 days before presentation. The patient also had a cryptococcal infection in the right lung and was receiving antifungal therapy with oral fluconazole at a dose of 0.2 g once daily. No recent changes had been made to her medication regimen. She denied the use of over-the-counter medications, herbal products, or dietary supplements. Physical examination at presentation showed that the patient was hemodynamically stable with no significant abnormalities. Family history was significant for recurrent episodes of hemolytic anemia in her elder brother.

Initial laboratory evaluation (**Supplementary Table 1**) revealed a white blood cell count of 5,840/mm³, hemoglobin of 3.2 g/dL, platelet count of 107,000/mm³, and a reticulocyte count of 31.43% (absolute reticulocyte count: 0.299 × 10^6^/mm³). Lactate dehydrogenase was markedly elevated at 1573 IU/L (upper limit of normal: 250 IU/L), with total bilirubin of 3.98 mg/dL (direct: 1.12 mg/dL; indirect: 2.86 mg/dL) and serum creatinine of 3.02 mg/dL. The direct Coombs test was positive. Serum C3 and C4 levels were decreased, and the κ/λ light chain ratio was increased. Bone marrow examination showed erythroid hyperplasia with a reduced myeloid-to-erythroid ratio. Molecular testing revealed no mutations in the α- or β-globin genes (excluding thalassemia).

**Figure 1:**
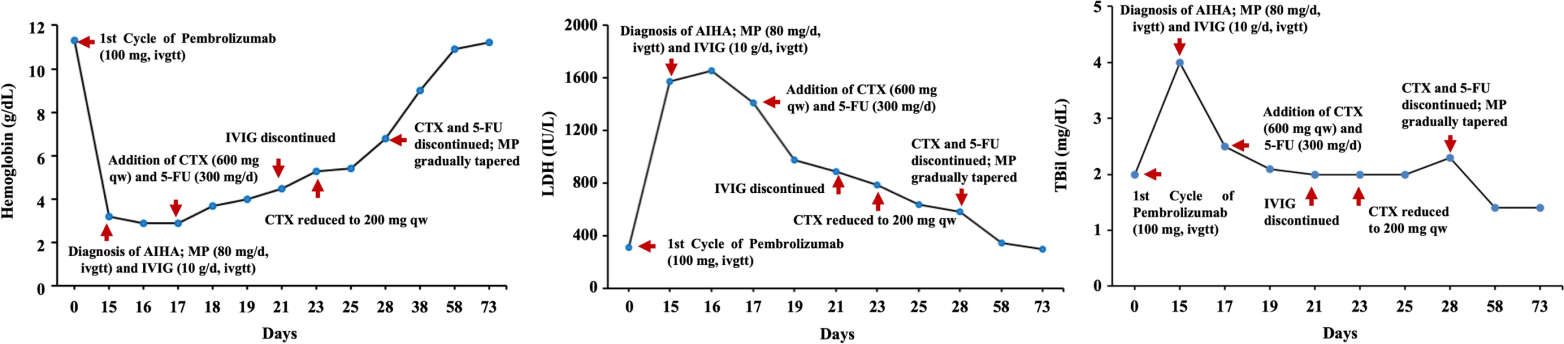
Hemoglobin, LDH, and total bilirubin changes over time during the first episode of AIHA. Cyclophosphamide (CTX), fluorouracil (5-FU), methylprednisolone (MP), intravenous immunoglobulin (IVIG).

**Figure 2:**
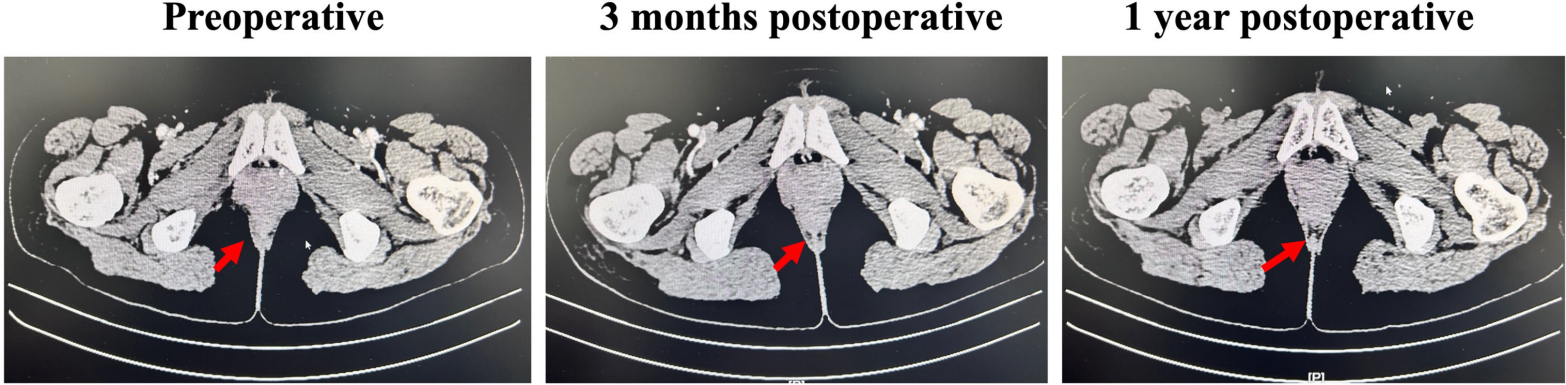
Abdominal CT images at various time points. Red arrows indicate the patient’s anal canal surgical area.

**Figure 3:**
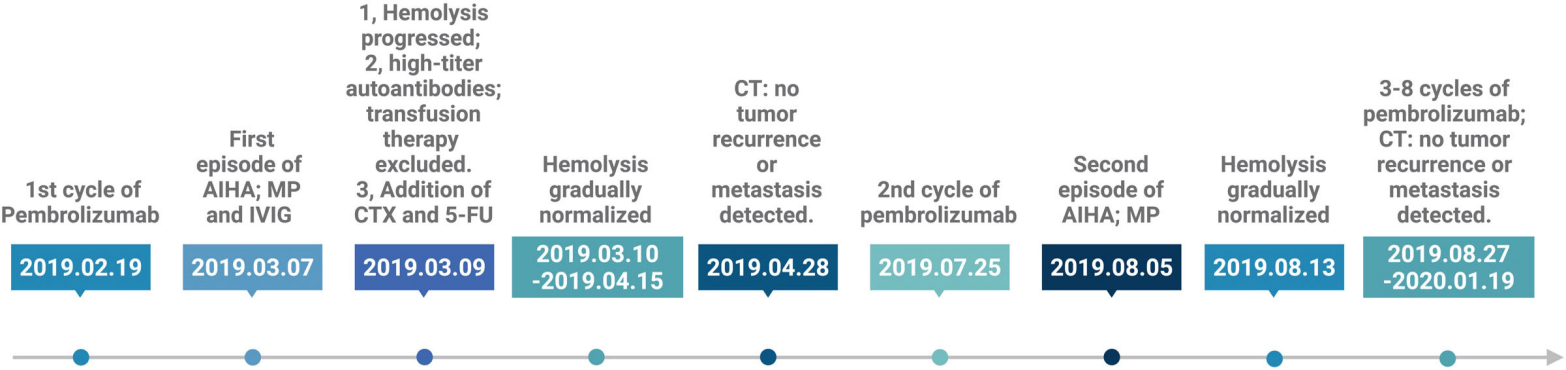
Timeline of the patient’s treatment course. Created in BioRender. Qin, Y. (2026) https://BioRender.com/9qetzab.

Based on the clinical presentation and laboratory findings, the patient was diagnosed with AIHA secondary to immunotherapy with pembrolizumab. Standard-dose corticosteroid therapy was initiated with intravenous methylprednisolone at a dose of 80 mg daily, in combination with intravenous immunoglobulin (IVIG) at 10 g daily for two consecutive days. Despite initial treatment, the patient’s condition deteriorated. She developed severe fatigue, marked lethargy, generalized jaundice, and oliguria. Repeat laboratory testing demonstrated a continued decline in hemoglobin, reaching a nadir of 2.9 g/dL. A multidisciplinary consultation was conducted, which concluded that compatibility testing revealed high-titer autoantibodies and a positive direct Coombs test. Given the high risk of exacerbating hemolysis, blood transfusion was not recommended. The consensus was to utilize a stronger immunosuppressive regimen. Accordingly, CTX was administered at a dose of 600 mg weekly, along with 5-FU at 300 mg daily, in combination with ongoing methylprednisolone and IVIG, to achieve better control of hemolysis. After one day of the modified treatment, the patient reported feeling less fatigue and examination revealed partial resolution of skin and scleral icterus. Laboratory results showed a hemoglobin level of 3.7 g/dL, lactate dehydrogenase (LDH) of 979 IU/L, total bilirubin of 2.13 mg/dL, direct bilirubin of 0.63 mg/dL, and indirect bilirubin of 1.5 mg/dL. Urinalysis revealed light yellow urine, urobilinogen 1+, and only traces of occult blood. Following four days of the combination therapy, the patient’s symptoms continued to improve. Laboratory findings showed hemoglobin at 4.5 g/dL, LDH at 886 IU/L, total bilirubin at 1.98 mg/dL, direct bilirubin at 0.58 mg/dL, indirect bilirubin at 1.40 mg/dL and urobilinogen 1 +. IVIG was then discontinued. After six days of the combination therapy, the patient’s symptoms improved significantly. Laboratory evaluation revealed hemoglobin at 5.3 g/dL, LDH at 786 IU/L, total bilirubin at 1.96 mg/dL, direct bilirubin at 0.61 mg/dL, and indirect bilirubin at 1.35 mg/dL. Urinalysis was negative for both urobilinogen and occult blood. The CTX dose was reduced to 200 mg weekly, and over the following days, the patient’s hematologic parameters continued to improve, with progressive declines in LDH and total bilirubin levels. After 11 days of treatment with methylprednisolone in combination with CTX and 5-FU, the latter were discontinued, and the steroid was gradually tapered. Over a total treatment course of approximately two months, the patient’s hemoglobin, LDH, and bilirubin levels returned to near-normal ranges (**Figure 1**). Pembrolizumab was withheld for approximately five months. At the strong request of the patient’s family and under close clinical monitoring, the patient was rechallenged with pembrolizumab. Ten days after re-administration, hemolytic anemia recurred, with laboratory findings showing hemoglobin of 8.8 g/dL, total bilirubin of 1.99 mg/dL, direct bilirubin of 0.71 mg/dL, and indirect bilirubin of 1.28 mg/dL. Intravenous methylprednisolone at a dose of 40 mg daily was immediately initiated and after eight days of therapy, her hemoglobin levels normalized. Immunotherapy was again suspended, but eventually the patient successfully completed cycles 3 through 8 of pembrolizumab with no recurrence of hemolytic anemia. During this period, serial imaging demonstrated good tumor control with no evidence of local recurrence or distal metastases (**Figure 2**). The timeline of the patient’s treatment course is shown in **Figure 3**. Clinical details of the patient are summarized in **Table 1**.

**Table 1:** Summary of the case information.

Age	52 years
Gender	Female
Family history	Brother with recurrent hemolytic anemia
Type of malignancy	Melanoma
Type of immunotherapy used	Pembrolizumab
Diagnosis	AIHA
Number of prior cycles	One and two
Number of occurrences of AIHA	Two
Treatment of 1st AIHA episode	Corticosteroid+IVIG+CTX+5-FU
Treatment of 2nd AIHA episode	Corticosteroid
Immunotherapy rechallenge	Yes
Total cycles of immunotherapy	Eight
Concomitant diseases	Cryptococcal infection of lung
Concomitant medications	Fluconazole
Outcome of concomitant conditions	Marked improvement

## Discussion

Melanoma is an aggressive malignancy arising from melanocytes and may occur in any site containing these cells, including the skin, various mucosal surfaces, and the eye (8). Globally, melanoma incidence continues to rise and accounts for a disproportionate share of mortality from skin cancer (9). Prognosis remains strongly stage-dependent, with limited efficacy of conventional therapies in advanced and mucosal disease; however, the introduction of immune checkpoint inhibitors has fundamentally changed melanoma management. Pembrolizumab, a PD-1 inhibitor, restores antitumor T-cell activity and has demonstrated significant survival benefits in advanced melanoma (10). In the phase III KEYNOTE-054 trial, adjuvant pembrolizumab significantly improved distal metastasis-free survival in resected stage III melanoma (11), while the KEYNOTE-716 study demonstrated durable tumor control in resected stage IIB/IIC disease (12). Although pembrolizumab remains an effective treatment option across melanoma stages, rare immune-related adverse events such as AIHA may occur.

AIHA is a rare but potentially life-threatening hematological disorder characterized by immune-mediated destruction of red blood cells (13). AIHA may be idiopathic or secondary to underlying conditions such as autoimmune diseases, infections, lymphoproliferative disorders, or drug exposure, including immune checkpoint inhibitors (14). With the increasing use of PD-1 inhibitors, immune-related hematologic toxicities have been increasing, although AIHA remains uncommon. The diagnosis of AIHA is based on clinical and laboratory evidence of hemolysis, including anemia, elevated lactate dehydrogenase, increased indirect bilirubin, reduced haptoglobin levels, and a positive direct antiglobulin (Coombs) test (13, 14).

Early recognition is critical, as delayed diagnosis may result in severe anemia and organ dysfunction. Once AIHA is diagnosed, identifying and removing the underlying cause is essential for successful disease control. Corticosteroids is the common choice of treatment for AIHA in many case reports because of its rapid suppression of autoantibody production and macrophage-mediated hemolysis (15). Blood transfusion can temporarily correct severe anemia but must be used with caution as it only provides a temporary benefit and may aggravate hemolysis in patients with high titers of autoantibodies (16). For steroid-refractory or relapsed AIHA, rituximab has proven effective by depleting CD20+ B cells and reducing pathogenic antibody production (17, 18 Dec 13). However, more therapeutic strategies for severe or refractory AIHA remain to be explored. Compared with previous studies, our case suggests that CTX combined with 5-FU constitutes a novel therapeutic option for severe AIHA in selected patients.

With the expanding use of immune checkpoint inhibitors, an increasing number of cases of immune-related autoimmune hemolytic anemia (irAIHA) have been reported following PD-1 inhibitor monotherapy, CTLA-4 inhibitor monotherapy, and combination PD-1/CTLA-4 therapy (19–23). Current evidence suggests that PD-1 blockade disrupts immune tolerance, leading to autoreactive T-cell activation and pathogenic autoantibody production, ultimately resulting in red blood cell destruction (24, 25). Recent systematic reviews and case-based analyses have further characterized the clinical features of irAIHA, demonstrating its variable presentation, frequent association with severe anemia, and a relatively high rate of direct antiglobulin test negativity (26). Importantly, recent multicenter data have provided important insights into the presentation and management of irAIHA, indicating that glucocorticoids are effective first-line therapy and that many patients can be safely re-challenged with immune checkpoint inhibitors after recovery (27).

Compared with previous reports, our case demonstrates several distinctive features. First, we introduced a novel immunosuppressive strategy combining CTX and 5-FU with corticosteroids and IVIG for the management of severe AIHA. CTX has long been recognized not only as a cytotoxic chemotherapeutic agent but also as a potent immunosuppressive drug (28). As highlighted in previous reviews, CTX exerts its immunosuppressive effects primarily by inhibiting the proliferation of rapidly dividing lymphocytes, including both T cells and B cells, through DNA cross-linking and the induction of apoptosis (29, 30). While CTX alone acts via this mechanism, the addition of 5-FU offers complementary immunomodulatory effects, particularly the reduction of regulatory T cells (Tregs) and myeloid-derived suppressor cells (MDSCs) as demonstrated by Lo Re et al. (31). Furthermore, this regimen was shown to be safe, feasible, and moderately active (31). The preclinical evidence from Ogiso et al. establishes the independent immunosuppressive capacity of both agents (32), while clinical studies in breast cancer confirm that CTX conbine 5-FU-containing regimens produce significant immune cell modulation (33). When used in combination, CTX suppresses the proliferation of autoreactive lymphocytes, while 5-FU may inhibit excessive immune activation, together providing synergistic immunosuppression. Based on these complementary mechanisms, the combination of CTX and 5-FU offers a biologically plausible therapeutic approach for patients with severe AIHA. Although our clinical experience suggests potential efficacy of this regimen, further validation in larger cohorts is necessary to confirm its safety and effectiveness. Additionally, studies comparing the therapeutic effects of combination therapy versus CTX monotherapy for AIHA would also be meaningful. If the immunosuppressive strategy combining CTX and 5-FU continues to prove effective, it could offer patients additional treatment options.

Our patient also underwent fluconazole treatment for a concurrent fungal infection. The hemolytic anemia was closely correlated with pembrolizumab exposure and resolved without interruption of the antifungal treatment, supporting pembrolizumab as the most likely causative agent. The patient also showed an uncommonly high titer of autoantibodies, indicating a substantial risk of transfusion-induced hemolytic exacerbation; thus, although transfusion rapidly alleviates anemia, it is only temporary and was inappropriate for this patient in the context of active AIHA. Decisions regarding transfusion should be carefully individualized, taking into account the severity of anemia, serologic findings, and the potential for further hemolytic deterioration. Following effective treatment, the previously detected transfusion-related antibodies disappeared in this patient. The precise mechanisms underlying this observation remain to be elucidated and merit further study. Lastly, a further distinctive feature of our patient was the unusually early occurrence of AIHA— developing after the first pembrolizumab treatment cycle and recurring at rechallenge; however, early recognition and prompt intervention resulted in successful control of hemolysis, permitting a subsequent rechallenge without recurrence and successful completion of immunotherapy cycles 3 to 8. This clinical course suggests a potential predisposition to immune-mediated hemolysis. Given the patient’s family history of recurrent episodes of hemolytic anemia in her elder brother, we speculate that an underlying genetic susceptibility may have contributed to the heightened risk of AIHA in this case.

## Patient perspective

To date, a number of case reports and clinical studies on AIHA have been published, and there is a general consensus regarding its clinical manifestations and diagnostic criteria. With respect to treatment, standard-dose corticosteroids remain the first-line therapy. For patients with severe disease or inadequate response to steroids, rituximab and blood transfusion have also been recognized as effective therapeutic options. However, there have been rare previous reports describing the use of a combination regimen of CTX and 5-FU for the correction of anemia in AIHA patients. Our case proposes a novel immunosuppressive strategy incorporating CTX and 5-FU in addition to corticosteroids and IVIG for the management of severe AIHA, which may offer patients additional treatment options. Furthermore, our case revealed the presence of high-titer autoantibodies in the patient, suggesting that blood transfusion could have triggered more severe hemolysis and even led to a fatal outcome. Consequently, transfusion therapy, despite being a timely and effective option, was excluded. During the treatment course, the patient experienced two episodes of AIHA. In the first episode, due to a lack of prior awareness and delayed intervention, the patient developed severe anemia and showed poor initial response to corticosteroids and IVIG. Only after intensification of immunosuppression with the combination of CTX and 5-FU was the anemia effectively controlled. In contrast, the second episode was detected promptly through proactive monitoring and was managed in a timely manner; as a result, the anemia was rapidly corrected with corticosteroid therapy alone. The patient subsequently completed cycles 3 through 8 of immune checkpoint inhibitor therapy without further recurrence of AIHA, emphasizing the importance of early diagnosis and appropriate treatment of AIHA in selected patients. Finally, in this case, AIHA occurred early, developing only two weeks after the first cycle of immunotherapy. The fact that the patient’s brother had a history of hemolytic anemia, suggests a potential genetic predisposition. This highlights the necessity of further investigation into the risk factors for AIHA, which may facilitate targeted preventive monitoring, early diagnosis, prompt intervention, and rapid correction of anemia. Although the patient was concurrently receiving antifungal medication during the first AIHA episode, the anemia was successfully resolved despite continued antifungal therapy, indirectly supporting that AIHA was induced by immunotherapy. Timely withdrawal of the causative agent, together with appropriate immunosuppressive treatment is the key to effective control of severe AIHA.

## Data Availability

The original contributions presented in the study are included in the article/**Supplementary Material**. Further inquiries can be directed to the corresponding authors.
