# Supplementary material for: Severe immune-related autoimmune hemolytic anemia induced by pembrolizumab: a case report with novel immunosuppressive strategy
Source: Front Immunol. 2026 May 8;17:1811425. doi: 10.3389/fimmu.2026.1811425 (PMC13194433; doi:10.3389/fimmu.2026.1811425)
Supplement: Supplementary file 1 [file DataSheet1.pdf]

Table S1 Initial laboratory findings at the time of diagnosis of AIHA

Laboratory parameters	Result	Reference range	Unit
Hemoglobin	3.2	11.5-15	g/dL
LDH	1,573	120-250	IU/L
Total bilirubin	3.98	0-1.35	mg/dL
Direct bilirubin	1.12	0-0.47	mg/dL
Indirect bilirubin	2.86	0-1.17	mg/dL
Reticulocyte count	31.43	0.59-2.07	%
Absolute reticulocyte count	0.299	0.022-0.106	$\times 10^6/mm^3$
Serum C3	<0.073	0.1-0.4	g/L
Serum C4	0.737	0.9-1.8	g/L
Coombs Test	Positive		
κ/λ light chain ratio	Increased		
Bone marrow smear	Erythroid hyperplasia, ↓ M/E		
Molecular testing	No α/β-globin genes mutations		
